# Role of Protein Kinase C-delta in regulating platelet activation and platelet-leukocyte interaction during sepsis

**DOI:** 10.1371/journal.pone.0195379

**Published:** 2018-04-04

**Authors:** Elisabetta Liverani, Mark J. Mondrinos, Shuang Sun, Satya P. Kunapuli, Laurie E. Kilpatrick

**Affiliations:** 1 Sol Sherry Thrombosis Research Center, Lewis Katz School of Medicine, Temple University, Philadelphia, Pennsylvania, United States of America; 2 Center for Inflammation, Translational and Clinical Lung Research, Department of Thoracic Medicine and Surgery, Lewis Katz School of Medicine, Temple University, Philadelphia, Pennsylvania, United States of America; 3 Department of Physiology, Lewis Katz School of Medicine, Temple University, Philadelphia, Pennsylvania, United States of America; Ann and Robert H Lurie Children's Hospital of Chicago, Northwestern University, UNITED STATES

## Abstract

Sepsis is characterized by an intense systemic inflammatory response activating a cascade of proinflammatory events resulting in leukocyte dysregulation and host tissue damage. The lung is particularly susceptible to systemic inflammation, leading to acute lung injury. Key to inflammation-induced lung damage is the excessive migration of neutrophils across the vascular endothelium. The mechanisms which regulate neutrophil activation and migration in sepsis are not well defined but there is growing evidence that platelets are actively involved and play a key role in microvascular permeability and neutrophil-mediated organ damage. We previously identified PKC-delta (PKCδ) as a critical regulator of the inflammatory response in sepsis and demonstrated PKCδ inhibition was lung protective. However, the role of PKCδ in sepsis-induced platelet activation and platelet-leukocyte interactions is not known. In this study, rats underwent sham surgery or cecal ligation and puncture (CLP) to induce sepsis. Following surgeries, a PKCδ inhibitor (200μg/kg) or vehicle (PBS) was administered intra-tracheally. At 24 hours post-surgeries, lung tissue, BAL fluid, and blood samples were collected. While sepsis caused thrombocytopenia, the remaining circulating platelets were activated as demonstrated by increased p-selectin expression, elevated plasma PF4, and enhanced platelet-leukocyte aggregate formation compared to Sham animals. Platelet activation was associated with increased platelet PKCδ activity. Inhibition of PKCδ attenuated sepsis-induced platelet activation, secretion and aggregate formation. Sepsis-induced thrombocytopenia was also significantly reduced and circulating platelet numbers were similar to sham animals. In the lung, sepsis induced significant influx of platelets and neutrophils and the development of lung injury. Administration of the PKCδ inhibitor decreased platelet and neutrophil influx, and was lung protective. Thus, PKCδ inhibition modulated platelet activity both locally and systemically, decreased neutrophil influx into the lung, and was lung protective. We demonstrate for the first time that PKCδ plays an important role in platelet activation and platelet-neutrophil interaction during sepsis.

## Introduction

Sepsis is a life-threatening organ dysfunction caused by a dysregulated host response to infection characterized by excessive neutrophil infiltration in the organs [1]. The lung is one of the first organs affected with pulmonary dysfunction often resulting in acute lung injury (ALI) [2–4]. The mechanisms that regulate neutrophil activation and migration in sepsis are not completely understood but there is mounting evidence that platelets are actively involved in neutrophil and endothelial activation and play an important role in neutrophil-mediated organ damage [5–8]. Platelets play a key role during hemostasis but they can also modulate the immune response through the release of pro-inflammatory mediators and through direct interaction with neutrophils and vascular endothelial cells, which can enhance neutrophil migration and contribute to microvascular permeability and organ damage [5–11]. In an animal model of sepsis, we demonstrated that anti-platelet therapy could decrease systemic inflammation and lung damage [12]. Furthermore, in septic patients there is increased platelet activation and adhesion to neutrophils and endothelium [13, 14]. All together, these data suggest that platelets play a key yet not well-defined role during sepsis.

To date, there are no specific pharmacologic therapies available that protect the lung from neutrophil-mediated damage [15–17]. We identified the Protein Kinase C isotype delta (PKCδ) as a critical regulator of the inflammatory response and an important signal transducer of multiple signaling pathways [18–22] suggesting PKCδ as an appropriate therapeutic target for the treatment of sepsis induced-lung injury [20, 23, 24]. While PKCδ plays an important role in platelet physiology [25–27], no studies have investigated the role of PKCδ in platelet function and platelet-neutrophil interaction during sepsis. To achieve this aim, we used a rat model of sepsis (cecal ligation and puncture (CLP)) to study platelet activation, secretion, and platelet-leukocyte interaction systemically and in the lungs. Our data show for the first time that PKCδ inhibition prevents sepsis-induced thrombocytopenia, modulates platelet functions and secretion in the blood as well as in the lung. Our studies demonstrate that PKCδ plays a relevant role in platelet-mediated activation in sepsis.

## Materials and methods

### Materials

All reagents, analytical grade, were obtained from Thermo Fisher Scientific (Waltham, MA) unless stated otherwise. Phospho Ser744/748 PKD2 was obtained from Cell Signaling Technologies (Beverly, MA). Phosphatase and protease inhibitor cocktails were from Sigma-Aldrich (St. Louis, MO). FITC-conjugated mouse anti-rat CD61 (Clone WT.5), PE-conjugated mouse anti-rat CD11b (Clone WT.5) and BD FACS^TM^ lysing solution were from BD Bioscience (San Diego, CA). Mouse anti-rat p-selectin antibody was purchased from Santa Cruz Biotechnology (Santa Cruz, CA). FITC-conjugated rabbit anti-goat was obtained from Thermo Fisher Scientific (Rockford, IL). Hoechst 33258 stain was from Life Technologies (Carlsbad, CA). Myeloperoxidase was from EMD Millipore (Temecula, CA).

### PKC*δ* Inhibitory peptide synthesis

As previously described [20], PKC*δ* activity was selectively inhibited by a peptide antagonist (PKC*δ*-Tat peptide) that consists of a sequence derived from the first unique region (V1) of PKC*δ* (SFNSYELGSL: amino acids 8–17) coupled via an N-terminal Cys-Cys disulfide bond to a membrane permeant peptide sequence in the human immunodeficiency virus TAT protein (YGRKKRRQRRR: amino acids 47–57 of TAT) [28]. This inhibitor targets docking domains and prevents translocation and substrate interaction [28]. This inhibitor targets the regulatory domain of PKCδ and not the ATP binding site so it is more specific than previously described PKC inhibitors [29, 30]. The peptide was synthesized by Mimotopes (Melbourne, Australia) and purified to >95% by preparative reverse phase high performance liquid chromatography. Extensive *in vitro* and *in vivo* studies have demonstrated that, when taken up by cells, the PKCδ-TAT peptide produces a unique dominant-negative phenotype that effectively inhibits activation of PKCδ, but not other PKC isotypes. [18, 28, 31]. Delivery of peptides to the lung by intra-venous or intra-peritoneal route can be difficult as these peptides are often rapidly cleared through uptake by the liver and kidney [32, 33]. Our previous studies have demonstrated that intra-tracheal (IT) administration of the PKCδ-TAT peptide decreased local and systemic inflammation and was lung protective in our rat model of sepsis induced by cecal ligation and puncture [20, 23, 24].

### Animal protocols

All animal handling and care adhered to the guide for the Care and Use of Laboratory Animals of the National Institutes of Health and they were approved by the Institutional Animal Care and Use Committee at Temple University School of Medicine (protocol #4488). Male Sprague-Dawley rats (200–250 g; Charles River, Boston, MA) were used in all experiments. Rats were acclimated for at least 1 week in a climate-controlled facility and given free access to food and water.

### Cecal ligation and puncture model

Sepsis was induced by the cecal ligation and puncture (CLP) method as described previously [20, 24]. All surgery was performed under anesthesia with isoflurane (2–4% induction in chamber and 1–2% maintenance in mask). A midline laparotomy was performed and the stalk joining the cecum to the large intestine will be ligated. The cecum was punctured twice with an 18-gauge needle on the anti- mesenteric border, stool expressed and the cecum returned to the abdomen. Sham controls underwent a laparotomy without cecal ligation or puncture. Following CLP or sham surgery, the abdominal incision was closed and the animals were orally intubated with a 16-gauge intravenous cannula and instilled with peptide (200μg/kg) or vehicle [phosphate-buffered saline (PBS)] and allowed to recover. The concentration of 200μg/kg was chosen based on our previous studies [23, 24] and in the work of others [31, 34].

Postoperative pain was managed by injection of 2 mg/kg bupivacaine (Marcaine, Hospira Inc., Lake Forest, IL) at the incision site prior to surgery, then every 8–12 hours postoperatively until euthanasia. Normal saline solution (50 ml/kg) was injected subcutaneously in all groups for fluid resuscitation. All animals in the study will be monitored continuously until recovery from anesthesia and then every 8–12 hours for signs of distress, pain or clinical problems based on unusual behavior (e.g., alterations in feeding, grooming, activity level). Rats were euthanized immediately if they demonstrated a moribund state as defined by labored breathing and immobility. We used 6–8 rats per group. The percentage of premature death in the CLP-operated group did not exceed 10%. Mortality was not observed in the sham-operated group.

At 24 hours post-surgery, the rats were anesthetized and blood samples were collected by cardiac puncture in 3.8% sodium citrate (10:1) for hematology studies (Hemavet^®^ Multispecies Hematology System, Drew Scientific, Inc. Oxford, CT) and the rats were euthanized by exsanguination. Blood samples for chemokine analysis were collected by cardiac puncture in heparin. Lungs were collected and fixed or frozen immediately in liquid nitrogen.

### Platelet isolation and western blotting analysis

Total blood collected in 3.8% sodium citrate (10:1) was centrifuged in polypropylene tubes at 22°C at 100×g for 10 min to obtain platelet rich plasma (PRP). PRP was centrifuged again at 400×g for 10 min. The platelet obtained were lysed in buffer containing 10 mM Hepes (pH 7.4), 150 mM NaCl, 5 mM EDTA, 1 mM Na-orthovanadate, 20 μM 4-(2-aminoethyl)-benzenesulfonyl fluoride, 1% Triton X-100, 5 μg/ml leupeptin, phosphatase and protease inhibitor cocktails. Protein concentrations of the cell lysates were determined by the BCA protein assay kit, according to the manufacturer’s instructions (Pierce). Proteins were separated on 4–12% SDS-PAGE gels at a protein concentration of 30 μg/lane. Protein kinase D2 (PKD2) activation was determined by immunoblotting of cell lysates using a phospho-specific antibody for PKD-2 (Ser744). Total protein content was determined using coomassie staining as previously reported [35]. After immune-detection, the membrane was washed twice with TTBS and then stained with 0.1% Coomassie (BioRad, Hercules CA) in methanol/water, (1:1) for 1 min, destained for 20min in acetic acid/ethanol/water, (1:5:4), washed with water and air-dried. The dry membrane was scanned and the staining density for the band at similar molecular weight as phosphor-PKD2 was analyzed with ImageJ software with an area outside the protein lanes defining the background.

### Lung fixation, processing for histology

The lungs were gravity-fixed with 10% neutral buffered formalin instillation into the airways; the trachea was then tied off, to maintain inflation during fixation. Lungs were fixed for 2 to 3 hours at room temperature, then held overnight in formalin at 4°C. After fixation, lungs were washed several times with PBS and stored in 70% ethanol at 4°C. Lung tissue samples were obtained from different locations (left and right lung) and depths (ventral and dorsal), to assess the uniformity of observed histological features and patterns of protein localization. Lung tissue was paraffin-embedded, cut into sections (8 to 10 μm thick), and stained with H&E or with specific antibodies for immune-histochemical detection of CD61 (platelets) and CD11b (leukocytes) [20, 23, 24]. H&E slides were analyzed by a second independent, blinded observer and representative images of five experiments are shown.

### Bronchoalveolar lavage fluid collection

In one group of animals, at 24 hours after surgery, bronchoalveolar lavage fluid (BALF) was obtained under anesthesia by inserting a cannula into the trachea and instilling 1.5 ml room temperature sterile PBS until the lungs were fully distended as described previously [20]. The fluid was withdrawn and saved. This process was repeated until a total of 4.5 ml PBS had been instilled. The samples were pooled for each animal and the volumes recorded. The percent BALF recovered was calculated and BALF volumes normalized. The BALF was centrifuged to remove cells, and supernatants were aliquoted and frozen at –70°C. Platelet count in the BALF was measured using the Hemavet^®^ Multispecies Hematology System (Drew Scientific, Inc. Oxford, CT).

### Platelet-leukocyte aggregate formation

Rat blood samples were incubated with FITC-conjugated anti-rat CD11b and PE-conjugated anti-rat CD61 for 20 minutes at 25°C. The reaction was stopped by adding BD FACS^TM^ lysing solution (1:10 in PBS). Samples were kept at 4°C prior to analysis. Flow cytometry was performed using a FACSCalibur analyzer and data were analyzed with FlowJo software as previously described [36–38]. Leukocytes were gated based on both cell shape and receptor expression (CD11b positive). Platelets and leukocytes were discriminated by forward and side light scatter, identified by their positive staining for PE-CD61 or PE-CD11b, respectively. Events double positive for FITC and PE identified platelet–leukocyte aggregates and were recorded as a percentage of a total of 10,000 gated leukocytes.

### Platelet Factor 4 (PF4)

Plasma was obtained after centrifugation of heparinized blood. PF4 was analyzed in plasma and BALF samples using a rat PF4 ELISA kit by TSZ ELISA (Waltham, MA) according to the manufacturers’ instructions. Results are expressed as International Units(IU)/ml.

### Myeloperoxidase peroxidation (MPO)

MPO enzymatic activity in lung tissue homogenates was measured as previously described [39], with modifications for use in a 96-well plate format [23]. In brief, lung tissue was homogenized in freshly prepared lysis buffer (0.5% hexadecyltrimethyl ammonium bromide in 50 mM potassium phosphate buffer) at a ratio of 0.1 g of wet tissue weight per milliliter of lysis buffer. Homogenates were cleared by centrifugation at 13,362 × *g* for 15 minutes at 4°C and placed on ice. On a per-well basis, the reaction mixture contained 284 *μ*l of 50 mM potassium phosphate buffer, 3 *μ*l of H_2_O_2_, and 3 *μ*l of 20 mg/ml o-dianisidine added to 10 *μ*l of sample to initiate the reaction (300 *μ*l total volume per well). Absorbance (460 nm) was measured over a 5-minute time course, and units of MPO activity were quantified using a standard curve.

### Fluorescence microscopy

Sections of rat lung tissue were deparaffinized, and antigen retrieval was achieved by microwaving the tissue slides for 4 min in citrate buffer pH 6.0. The slides were washed and incubated with FITC-conjugated mouse anti-rat CD61 for analysis of platelet sequestration in the lung. Alternatively, slides were incubated with both FITC-conjugated mouse anti-rat CD61 and PE-conjugated mouse anti-rat CD11b to detect platelet-leukocyte interaction in the lung. The slides were mounted in Vectashield with DAPI (DNA stain) and imaged using fluorescence microscopy. The Image J cell counting program was used to determine fluorescence mean. Analysis of random fields (n = 6) from each section (n = 3 sections/animal) was performed by a second independent, blinded observer.

### P-selectin expression

Washed platelets were incubated with goat anti-rat p-selectin antibody for 1 hour at room temperature. Cells were washed and incubated with FITC-conjugated rabbit anti-goat for 1 hour at room temperature and the platelets were fixed in 1% paraformaldehyde. Flow cytometry was performed using a FACSCalibur analyzer and data analyzed with FlowJo software. Data are recorded as percentage of cells expressing p-selectin over a total of 10,000 cells analyzed.

### Statistics

Results are expressed as means ± S.E.M. Data were analyzed by one-way analysis of variance for multiple comparisons followed by Tukey-Kramer multiple comparisons post-test to evaluate the significance between experimental groups if analysis of variance indicated a significant difference. Student’s t tests were used to assess individual pairwise comparisons. Differences were considered significant when *P* <0.05.

## Results

### PKCδ inhibitor decreases sepsis-mediated activation of platelet PKCδ

**Fig 1:**
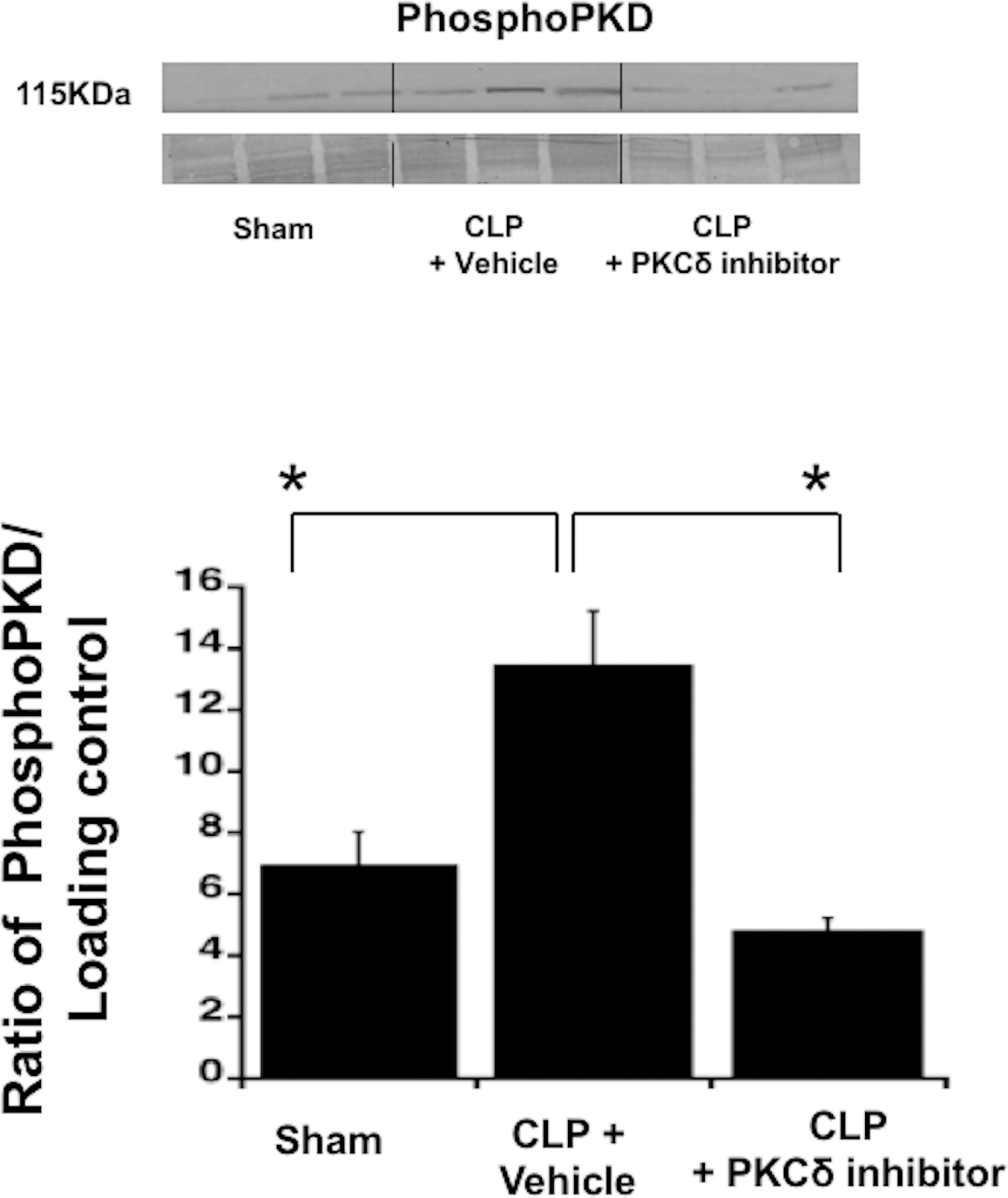
PKCδ inhibitor decreases sepsis-mediated activation of platelet PKCδ. Rat platelets were isolated from blood samples of the following groups: Sham, vehicle-treated CLP and CLP treated with with PKCδ inhibitor. PKD2 phosphorylation was measured by Western Blotting analysis using phospho-specific antibody against PKD2. A representative image (top) and the ratio between phospho-PKD2 and loading control band (bottom) are shown. (*p < 0.05; Sham versus CLP plus vehicle; CLP plus vehicle versus CLP plus PKCδ inhibitor, n = 5).

We first determined whether platelet PKCδ was activated in response to sepsis. Previous studies from our group identified Protein kinase D2 (PKD2) as a specific substrate for PKCδ but not for other PKC isoforms in platelets [40]. Thus, phosphorylation of PKD2 is a marker of PKCδ activation in platelets. Platelets were isolated from blood samples obtained 24 hours post sham or CLP surgery. As shown in Fig 1, 24 hours post CLP surgery, there were significant increases in PDK2 phosphorylation compared with the Sham control. In contrast, when rats were treated with the PKCδ inhibitor following CLP surgery, PKD2 phosphorylation was significantly diminished as compared to untreated septic rats (Fig 1). These data show that administration of the PKCδ inhibitor was able to limit sepsis-induced platelet PKCδ activation.

### PKC*δ* inhibition attenuated sepsis-induced thrombocytopenia and decreased sepsis-induced platelet activation in the blood

**Fig 2:**
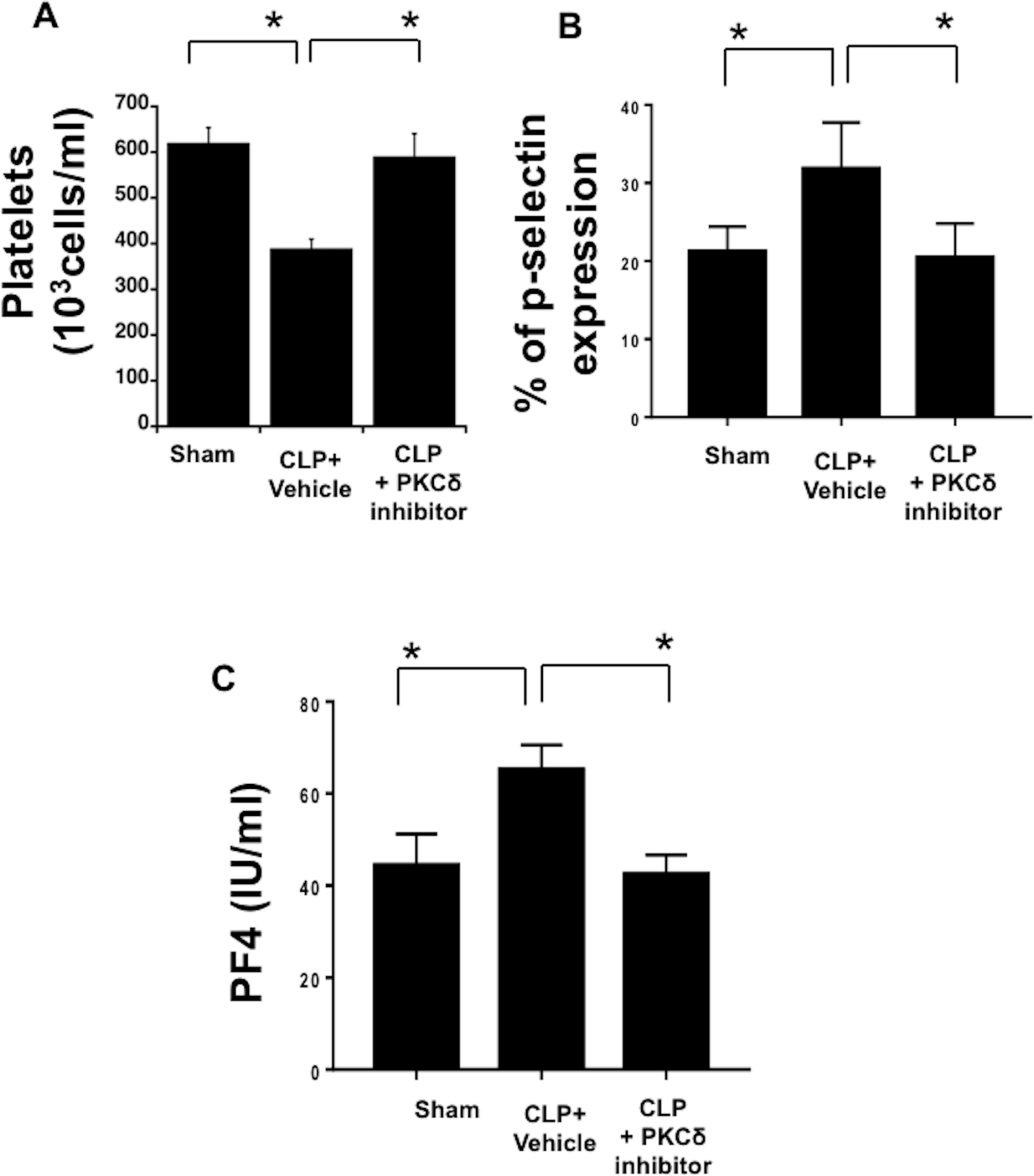
PKC*δ* inhibition prevented sepsis-induced thrombocytopenia and decreased sepsis-induced platelet activation in the blood. (**A**) Levels of circulating platelets were analyzed in Sham, vehicle-treated CLP and CLP treated with PKCδ inhibitor animals using a Hemavet^®^ Multispecies Hematology System (*p < 0.05; Sham versus CLP; CLP versus CLP plus PKCδ inhibitor, n = 6) (**B**) P-selectin expression on platelet surfaces was evaluated in Sham, CLP plus vehicle and CLP treated with PKCδ inhibitor using flow cytometry (*p < 0.05; Sham versus CLP plus vehicle; CLP plus vehicle versus CLP plus PKCδ inhibitor, n = 6) (**C**) Plasma levels of PF4 was evaluated in Sham, CLP plus vehicle and CLP treated with PKCδ inhibitor by ELISA (*p < 0.05; Sham versus CLP plus vehicle; CLP plus vehicle versus CLP plus PKCδ inhibitor, n = 6).

As thrombocytopenia is often observed clinically in septic patients, as well as in animal models of sepsis [41, 42], we investigated whether PKCδ inhibition can alter sepsis-induced thrombocytopenia. We first analyzed circulating platelet counts in the whole blood of Sham, CLP plus vehicle and CLP plus PKCδ inhibitor groups (Fig 2A). Thrombocytopenia was observed in septic animals 24 hours post-surgery, compared with Sham controls (*P* < 0.05, Sham vs CLP). Treatment with the PKCδ inhibitor significantly attenuated the thrombocytopenia (*P* < 0.05, CLP plus vehicle vs CLP + PKCδ inhibitor) and circulating platelet levels were similar to blood levels measured in sham surgery animals (P = NS sham vs CLP + PKCδ inhibitor). Platelet p-selectin surface expression was analyzed by flow cytometry to determine platelet activation (Fig 2B). In septic rats, p-selectin expression was increased, compared with the Sham control (*P* < 0.05, Sham vs CLP plus vehicle). In contrast, in animals treated with the PKC*δ* inhibitor, p-selectin expression was significantly reduced compared with vehicle-treated septic rats (*P* < 0.05, CLP plus vehicle vs CLP + PKCδ inhibitor). PF4, an important neutrophil chemoattractant, regulates neutrophil recruitment and tissue damage during inflammation [43]. This chemokine is released exclusively from the alpha-granules of activated platelets [44] and megakaryocytes [43]. PF4 is elevated in plasma samples of septic patients [45]. To ascertain the level of PF4 in our sepsis model and the impact of PKCδ inhibition, we next analyzed the levels of PF4 in plasma samples by ELISA (Fig 2C). PF4 levels were significantly increased in plasma samples from septic rats (*P* < 0.05, Sham vs CLP plus vehicle), but when rats were treated with the PKCδ inhibitor, PF4 secretion was significantly reduced as compared to septic rats treated with vehicle (*P* < 0.05, CLP plus vehicle vs CLP + PKC*δ* inhibitor). These data indicate that PKCδ inhibition can modulate sepsis-induced thrombocytopenia, platelet activation, and PF4 secretion.

### PKCδ inhibition decreased sepsis-induced platelet-leukocyte aggregate formation

**Fig 3:**
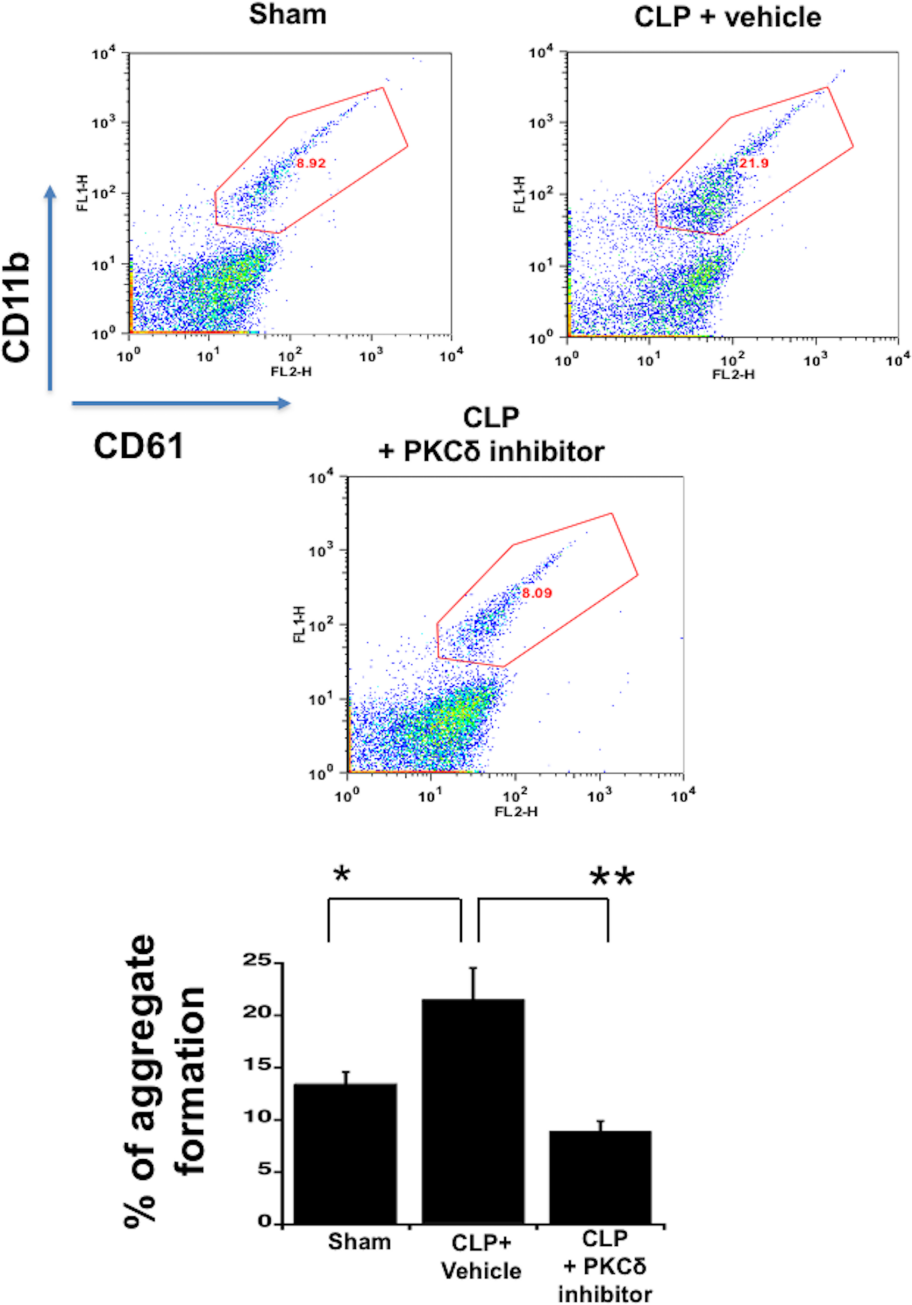
Treatment with the PKCδ inhibitor decreased sepsis-induced platelet-leukocyte aggregate formation. Blood samples were incubated with antibodies against CD61 (platelet marker) and CD11b (leukocyte marker). Activated leukocytes were gated based on CD11b expression and cell shape and data were analyzed as a percentage of aggregates expressing both CD61 and CD11b (**A**) Representative dot plot images of percentage of neutrophil-platelet aggregates in rat blood samples of Sham, CLP plus vehicle and CLP plus PKCδ inhibitor. (**B**) Graph showing the percentage of aggregate formation of the data represented in (A) (*p <0.05 sham versus CLP, **p < 0.01; CLP plus vehicle versus CLP plus PKCδ inhibitor, n = 4).

To determine whether PKC*δ* inhibition could alter the systemic interaction between platelets and leukocytes, we analyzed platelet-leukocyte aggregate formation in whole blood (Fig 3). At 24 hours post CLP surgery, there was a significant increase in platelet-leukocyte aggregate formation as compared to sham surgery rats (*P* < 0.05, Sham vs CLP plus vehicle) which was significantly reduced in rats treated with the PKCδ inhibitor following CLP surgery (*P* < 0.01, CLP plus vehicle vs CLP + PKCδ inhibitor). These data suggest an important role for PKCδ in regulating the interaction of circulating platelets with leukocytes during sepsis.

### Treatment with the PKCδ inhibitor altered neutrophil and platelet infiltration into the lungs of septic rats

**Fig 4:**
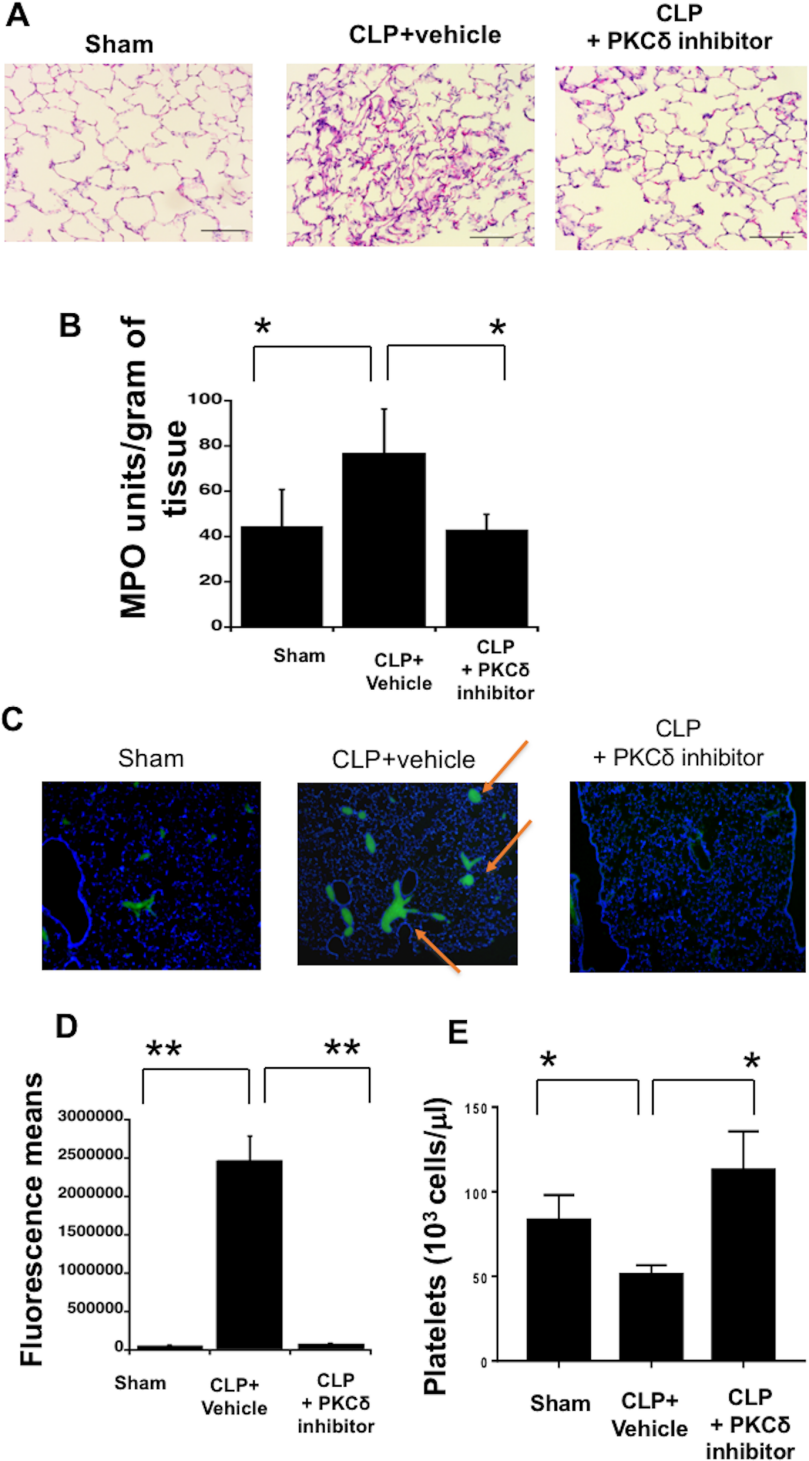
The effect of PKCδ inhibition on sepsis-induced lung injury, neutrophil sequestration and platelet influx in the lung and BALF. (**A**) Photomicrographs of hematoxylin- and eosin-stained tissue sections were obtained 24 hours after Sham or CLP surgery. Representative images of lung tissue specimens are shown for sham, CLP and CLP plus PKCδ inhibitor (Magnification 20; *n* = 5). (**B**) MPO analysis was performed in lung samples of sham and CLP rats. Values are expressed as units per gram of tissue (*p < 0.05; sham versus CLP plus vehicle and CLP plus vehicle versus CLP plus PKCδ inhibitor, n = 6). (**C**) Platelet accumulation in the lung was studied thought fluorescence microscopy (blue) DAPI; Green CD61, platelets, n = 4). (**D**) Fluorescence mean of CD61 staining values (**p<0.01 Sham versus CLP plus vehicle and CLP plus vehicle versus CLP plus PKCδ inhibitor, n = 4). (**E**) Platelet counts were analyzed in the BAL fluid of Sham and CLP animals. Cells were counted using a Hemavet^®^ Multispecies Hematology System (*p < 0.05; Sham versus CLP plus vehicle and CLP plus vehicle versus CLP plus PKCδ inhibitor, n = 6).

Sepsis produces systemic inflammation, which leads to the influx of neutrophil into the lung and the development of acute lung injury. Recent studies suggest that platelet-neutrophil interactions play an important role in sepsis-induced lung injury [46], however the role of PKCδ in platelet recruitment to the lung has not been studied. In agreement with our previous studies [20, 23, 24], at 24 hours post-CLP surgery there is significant lung injury and leukocyte influx as compared to rats that had undergone sham surgery (Fig 4). In the sham surgery animals, H&E staining showed normal lung architecture with minimal inflammatory cell infiltrates. Lungs from CLP surgery rats who received vehicle had significant evidence of inflammation and pulmonary injury. Septic animals who received the PKCδ inhibitor had limited infiltrates and preserved lung architecture. We also analyzed MPO activity as a marker for inflammatory cell infiltration. As expected, 24 hours post CLP surgery, there was a significant increase in lung MPO as compared to sham surgery animals (P<0.05 sham vs CLP plus vehicle) which was attenuated by PKCδ inhibitor treatment (Fig 4B, *P* < 0.05, CLP plus vehicle vs CLP + PKCδ inhibitor).

Platelet infiltration was analyzed by immunofluorescence staining of lung tissue and platelet counts in the BALF. Platelet influx into the lung was detected by measuring CD61 positive cells. Twenty-four hours post CLP surgery there was a significant increase in CD61 positive cells in the lung as compared to sham surgery animals (Fig 4C and 4D, *P* < 0.01, CLP plus vehicle vs Sham). Conversely, following PKCδ inhibitor treatment, the number of CD61 positive cells was significantly decreased compared with the CLP group (*P* < 0.01, CLP plus vehicle vs CLP + PKCδ inhibitor) as indicated by the red arrows in the figure. The increased sequestration of platelets in the lung is reflected by a decrease in platelet numbers in the BALF of septic rats as compared to sham animals (Fig 4E, *P* < 0.05, CLP plus vehicle vs Sham). BALF platelet numbers increased to control levels following PKCδ inhibitor treatment. These data indicate that during sepsis, platelets are sequestrated in the tissue, rather than in the BALF. Interestingly, PKCδ inhibition prevented platelet sequestration in the lung.

### Treatment with the PKCδ inhibitor alters platelet secretion in the lungs of septic rats

**Fig 5:**
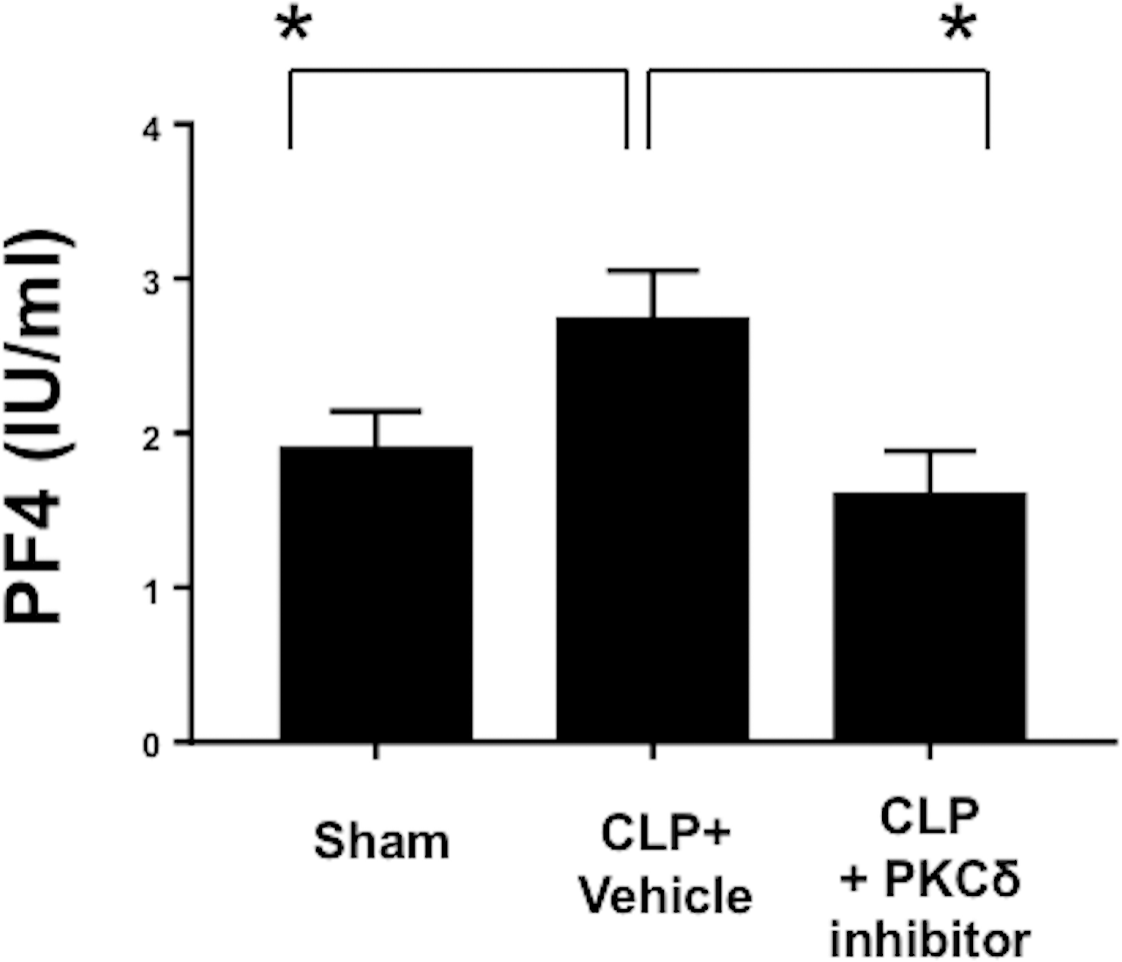
Treatment with the PKCδ inhibitor alters platelet secretion into the lungs of septic rats. PF4 concentration in the BALF was analyzed by ELISA. Data are expressed as International Units (IU)/ml (*p < 0.05; Sham versus CLP plus vehicle and CLP plus vehicle versus CLP plus PKCδ inhibitor, n = 6).

To determine whether platelet secretion was altered in the lung during sepsis, we analyzed PF4 content in the BALF (Fig 5). There was an increase in PF4 secretion, in septic rats (*P* < 0.05, CLP plus vehicle vs Sham), which was significantly decreased in PKCδ inhibitor treated septic rats as compared to vehicle treated septic rats (*P* < 0.05, CLP plus vehicle vs CLP + PKCδ inhibitor). Thus, in sepsis, systemic inflammation leads to platelet activation, sequestration in the lungs and local platelet secretion. PKCδ inhibition prevents platelet activation and sequestration in the lungs.

### Sepsis-induced platelets/leukocytes aggregate formation is prevented in the lungs of PKCδ inhibitor treated rats

**Fig 6:**
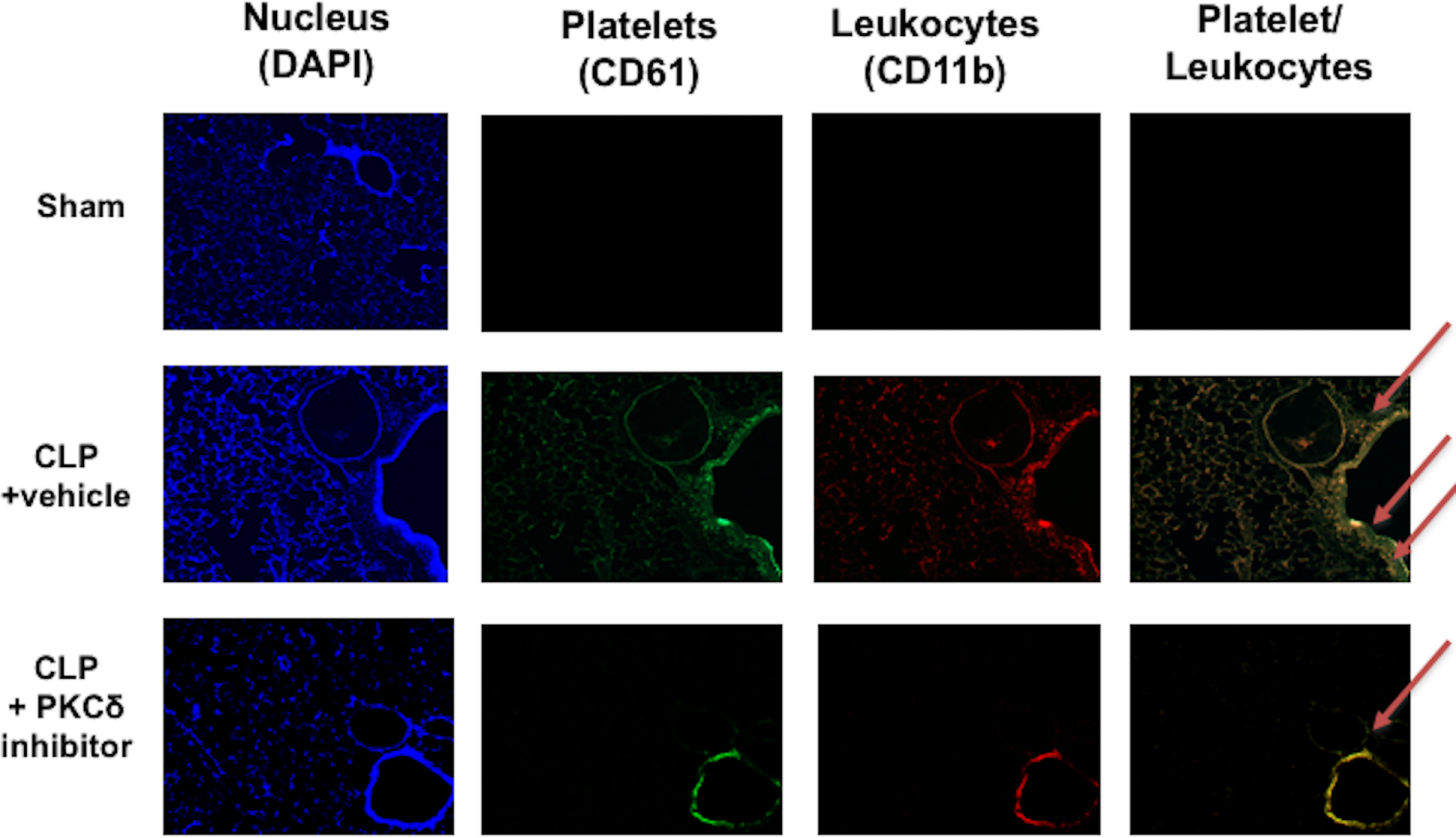
Platelet-leukocyte co-localization in the lungs. Lung tissue were co-stained with FITC-conjugated anti-CD61 (platelets) and PE-conjugated anti-CD11b (leukocytes) in Sham and CLP plus vehicle rats. Nuclei were labelled with DAPI. The first column shows nuclei staining, the second column platelet sequestration in the lung. The third column shows leukocyte infiltration in the lung tissue, while the last column shows both platelet and leukocyte content (red arrows). Images are representative of 4 independent experiments.

To analyze how PKCδ inhibition influences platelet/leukocyte interactions in the lung, we stained lung tissue for CD61 (platelet marker) and CD11b (leukocyte marker) (Fig 6). In Sham rats, the lungs did not show significant cell infiltration (Fig 6, top panels), while in septic animals both platelets and leukocytes infiltration was observed (Fig 6, middle panels). Co-localization was observed suggesting leukocyte-platelet aggregation in the lungs as well as in the periphery during sepsis, as indicated by red arrows in the figure. In contrast, PKCδ inhibitor treated rats had decreased levels of platelet and neutrophil interaction in the lungs, suggesting a decrease in aggregation (Fig 6, bottom panels).

## Discussion

Platelets play an important role in hemostasis but they are also increasingly recognized for their ability to modulate immune responses through cell-cell interaction and secretion [5]. Importantly in sepsis, neutrophil recruitment to the lung is platelet dependent and platelet depletion results in decreased lung damage in models of acute lung injury [46]. PKCδ has a crucial role in platelet functions during hemostasis, including secretion, but no studies have investigated the role of PKCδ activity in platelets during sepsis. We show for the first time that PKCδ is activated in platelets during sepsis and that PKCδ inhibition decreases sepsis-induced platelet activation and their interaction with leukocytes both systemically and in the lung in the CLP model of sepsis in rats. These data suggest a role for PKCδ in the regulation of platelet activity during sepsis and this modulation of platelet activity was associated with reduced neutrophil migration into the lung and decreased lung injury.

Septic patients often develop thrombocytopenia and this has been identified as an independent predictor of severity and mortality [47], similarly to what is observed in a murine model of sepsis [48]. Our data show that while septic animals develop thrombocytopenia 24 hours post-surgery, PKCδ inhibition was able to maintain a platelet count comparable to sham animals. The increase in circulating platelets in septic rats treated with the PKCδ inhibitor may be the result of multiple factors. Sepsis can increase thrombotic events and/or platelet infiltration in the organs [49] hence PKCδ inhibition could decrease thrombocytopenia by preventing thrombosis and platelet infiltration. In line with this mechanism, our studies demonstrate increased lung sequestration of platelets accompanied by decreased platelet numbers in the BALF during sepsis. Interestingly following treatment with the PKCδ inhibitor, platelet levels in the BALF increased to levels observed in sham animals. Alternatively, PKCδ inhibition may increase platelet formation and/or decrease platelet clearance. Indeed, in previous studies from our group, we found that megakaryopoiesis was enhanced in PKCδ null mice and PKCδ null mice recovered faster from an immune-mediated thrombocytopenia as compared to wild type mice[50], suggesting that PKCδ is an important regulator of platelet formation and PKCδ inhibition could also increase megakaryopoiesis during sepsis. Thus, it would be interesting to determine whether this inhibitor can be effective in other models of thrombocytopenia.

Circulating platelet functions are altered in septic patients and platelet activation readouts have been suggested as important biomarkers for the development of sepsis complications and may be an indicator of prognosis [49]. In particular, p-selectin levels in plasma samples of septic patients were increased compared to healthy controls [45], indicating that the regulation of p-selectin expression may play a role in sepsis. In our animal model of sepsis, p-selectin expression was significantly increased in septic rats, while treatments with the PKCδ inhibitor attenuated sepsis-induced p-selectin expression.

P-selectin binds PSGL-1 on leukocytes, activating the leukocytes and promoting their infiltration into the inflamed tissue [51]. Previous studies have demonstrated that platelet depletion attenuates neutrophil recruitment to the lungs in the CLP model of sepsis [46]. Our data show that the sepsis-induced platelet-leukocyte aggregate formation was reduced upon PKCδ inhibition both systemically and in the lungs. This reduction in platelet sequestration in the lung was associated with decreased in neutrophil infiltration and a reduction in tissue damage. This is in line with our previous observations where P2Y_12_ antagonism as well as P2Y_12_ deficiency reduced p-selectin expression, platelet-leukocyte aggregation in the blood and the lung tissue, and reduced lung injury in the CLP model of sepsis [12]. However, during bacterial inflammation [52], platelet depletion caused increased mortality and decreased bacterial clearance, compared with non-depleted mice. These data together suggest that modulating platelet functions and platelet-leukocyte interaction may play a key factor in the development of lung injury in sepsis. As PKCδ inhibition prevents TNF-induced adhesion [18] and oxygen radical production [19] in neutrophils *in vitro*, a direct effect of this inhibitor on neutrophils is also possible. However, in this paper we demonstrate a direct effect on platelets, as we show that sepsis induced platelet PKD2 phosphorylation, a known substrate of PKCδ in platelets [40]. Further, treatment with the specific PKCδ inhibitor decreased PKD2 phosphorylation as well as platelet p-selectin expression indicating a direct effect on platelets in this sepsis model. It would be interesting to obtain further information about platelet-leukocyte interaction in the lung using intra-vital microscopy as previously done in the liver [53].

PF4 is the most abundant protein contained in platelet *α*-granules and it is released by activated platelets [54] and megakaryocytes [43]. PF4 is important not only in hemostasis, but also during inflammation [55]. In our model of sepsis, PF4 levels were increased in both the plasma and the BALF of septic animals compared with the sham control. Interestingly, PKCδ inhibition decreased PF4 levels both systemically and in the lung. Previous studies have shown that in PF4 null mice, lung functions are preserved in a murine model of ALI [55], indicating that a decrease in this chemokine levels can protect from lung injury. Furthermore, PF4 promotes neutrophil granule release and adhesion to endothelial cells [56]; as well as phagocytosis, chemotaxis, and generation of reactive oxygen species in leukocytes [56]. Indeed in a LPS-induced model of ALI, neutrophil recruitment to the lungs was shown to be dependent on PF4 [57]. Considering these data together, alongside with reduced p-selectin expression and platelet-leukocyte cell-cell interactions, decreased levels of PF4 could also be the mechanism through which platelets modulate neutrophil infiltration in the lung and prevent injury.

PKCδ has been identified in different signaling pathways in platelets and it can either be a positive or negative regulator of platelet activation and secretion [27, 56, 57]. In some pathways such as through thrombin and collagen receptors, PKCδ is directly involved [27, 56, 57], while through other pathways such as TLR-4 and ADP receptor activation [58, 59], PKCδ may be involved indirectly. The integration of these multiple signaling pathways have not yet been delineated during sepsis. However, our studies demonstrate that inhibition of PKCδ signaling in platelets during sepsis can modulate cell functions systemically and locally and it results in an overall protective effect. In the future, it would be interesting to evaluate whether PKCδ inhibition can also regulate thrombotic events in sepsis as well as inflammation levels. In conclusion, PKCδ inhibition modulates platelet activity both systemically and locally, decreased neutrophil influx into the lung and was lung protective. Thus, PKCδ plays an important role in platelet activation during sepsis.

## Supporting information

S1 Data (XLSX)
